# Oxidative Response and Antioxidative Mechanism in Germinating Soybean Seeds Exposed to Cadmium

**DOI:** 10.3390/ijerph9082827

**Published:** 2012-08-08

**Authors:** Shiyong Yang, Jianchun Xie, Quanfa Li

**Affiliations:** 1 Section of Ecology, School of Life Sciences, Anhui Normal University, Wuhu 241000, China; Email: lqfirris@mail.ahnu.edu.cn; 2 School of Environmental Science and Engineering, Anhui Normal University, Wuhu 241000, China; Email: xjch77@tom.com

**Keywords:** cadmium, soybean, oxidative stress, antioxidative response, bioindicator

## Abstract

Seeds of soybean (*Glycine* *max* L.) exposed to 50 mg/L (Cd50), 100 mg/L (Cd100) and 200 mg/L (Cd200) cadmium solution for 24, 48, 72 and 96 h were examined with reference to Cd accumulation, oxidative stress and antioxidative responses. Soybean seeds accumulated Cd in an exposure time-and dosage-dependent manner. FRAP (ferric reducing ability of plasma) concentration, GSH/hGSH content, and GST activity showed a pronounced exposure time-dependent response. Cd100 enhanced FRAP concentration in germinating soybean seeds as compared to Cd50 treatment after 24 h exposure. Cd200 however increased statistically GST activities after 72 and 96 h exposure. Under all Cd dosages, GSH/hGSH concentrations were depressed with increasing exposure time. Reduction of GSH/hGSH content and concomitant increase of GST activity suggested a possible participation of GSH into GSH-Cd conjugates synthesis. MDA content is a potential biomarker for monitoring Cd phytotoxicity because it responds significantly to treatment dosage, exposure time and dosage × exposure time interaction. Increase of proline content may be a response to acute heavy metal toxicity in soybean seeds.

## 1. Introduction

Cadmium (Cd) is a non-essential trace element without any known physiological function. It enters the ecosystem chiefly as the consequence of anthropogenic activities as well as mineralization processes of rocks enriched with Cd. The accumulation of Cd in soil is dangerous to plants because this metal shows phytotoxicity even at low doses and may impair human health via the food chain. Although Cd content in natural soils is very low, industrial, agricultural and municipal activities have increased its accession to agricultural soils. Cadmium content in phosphate fertilizer can be up to 200 mg/kg [1], and sewage sludge contains as much as 815 mg/kg Cd [2].

Cd leads to a diverse of morphological, physiological, biochemical and structural changes in growing plants. Such changes are the probable consequences of oxidative stress, namely formation of reactive oxygen species (ROS), increase of lipid peroxidation, and decrease in the activities of enzymatic and non-enzymatic antioxidants [3,4,5,6]. The ecotoxicological effects of Cd on a variety of adult plants and seedlings have been extensively studied, whereas the toxicity of Cd in the early stage of ontogenesis, e.g., during seed germination, has received relatively less attention [5,6,7].

Soybean (*Glycine* *max* L.) is an important crop worldwide. Reduction of biomass production and nutritional quality has been observed in crops grown on metal-contaminated soils. Previous studies in soybean indicated that 0–2.0 mM Cd severely inhibited plant growth, and in response to Cd exposure both the amount of non-enzymatic antioxidants and activities of enzymatic antioxidants were elicited [8]. Oxidative stress and enhanced root noodle senescence were observed in soybean plants subjected to Cd exposure [9]. A heme oxygenase also implicated in the protection of soybean leaves against Cd stress [10]. To the best of our knowledge, however, only a few studies have ever focused on the effects of Cd exposure on the early stage of ontogenesis in germinating soybean seeds [11]. This present study attempted to investigate the impact of imbibition with Cd on proline content, oxidative stress as indicated by lipid peroxidation, and antioxidant mechanism as presented by FRAP, GSH content and GST activity, in germinating soybean seeds. The present work shall help to explain the oxidative and antioxidative response of germinating seeds subjected to cadmium treatment.

## 2. Results and Discussion

### 2.1. Results

#### 2.1.1. Cd Concentration in Germinating Seeds

Cadmium concentration in germinating soybean seeds increased significantly with increasing Cd dosage (Table 1, F = 12.11, *p* < 0.001). Cd concentration in seeds treated by Cd50 was 3.11–14.74-fold, while those of Cd100 and Cd200 treatment were 3.57–26.82 and 6.13–42.23-fold higher than those in controls, respectively, depending on exposure time. Interestingly, At Cd200, Cd concentration in seeds increased after 48 h exposure, but it declined after 72 h exposure. Analysis also suggested that there was an interaction between exposure time and treatment concentration, in other words, Cd concentration in germinating seeds is affected by both treatment duration and Cd dosage (F_9,__48_ = 30.49, *p* < 0.001). At treatment Cd50 and Cd100, there was significant positive correlation between exposure time and Cd concentration in soybean seeds (r = 0.76, *p* = 0.004, and r = 0.90, *p* < 0.001, respectively).

**Table 1:** Cd concentration in germinating soybean seeds after 24, 48, 72 and 96 h Cd exposure (μg/g).

Cd concentration (mg/L)	Exposure time (h)			
	24	48	72	96
0	1.67 ± 0.25a	1.67 ± 0.25a	1.67 ± 0.25a	1.67 ± 0.25a
50	6.88 ± 0.64b	8.52 ± 3.06ab	11.94 ± 2.07a	26.28 ± 11.4b
100	7.64 ± 1.36bc	16.58 ± 6.22b	40.56 ± 13.2b	46.46 ± 7.05c
200	11.9 ± 2.31c	31.86 ± 5.82c	72.19 ± 13.9c	33.94 ± 2.76bc

Note: Values followed by lower case letters are significant at *p* < 0.05.

#### 2.1.2. Effects of Cd on Total Antioxidant Capacity

**Figure 1:**
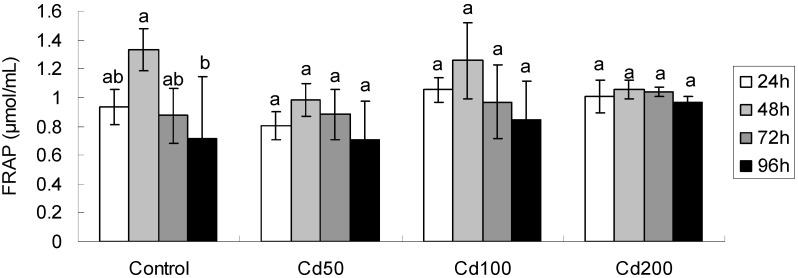
The total antioxidant capacity (FRAP) in germinating soybean seeds after different periods of exposure. All values are mean ± SD (n = 4). Values followed by lower case letters are significant at *p* < 0.05.

Exposure time affected significantly FRAP concentration (*p* < 0.001, Table 2). For all treatments, FRAP concentrations were negatively correlated with exposure time, though the effect was non-significant. The concentrations increased after 48 h exposure, but declined afterwards (Figure 1). During 24 h exposure, FRAP concentration at Cd100 was significantly higher than that at Cd50 (F = 4.42, *p* = 0.026), while during 48 h exposure, the value at Cd0 (control) was significantly higher than that at Cd50 treatment.

**Table 2:** Results of two-way analysis of exposure time and Cd concentration on the concentration of GSH, MDA and proline, total antioxidant capacity (FRAP) and GST activity of germinating soybean seed extract.

Effect	FRAP	MDA	GSH	Proline	GST
Time	F_3,__48_ = 9.15 **	F_3,__48_ = 52.55 **	F_3,__48_ = 59.67 **	F_3,__48_ = 113.8 **	F_3,__48_ = 4.40 **
Conc	F_3,__48_ = 2.51	F_3,__48_ = 17.72 **	F_3,__48_ = 2.02	F_3,__48_ = 9.31 **	F_3,__48_ = 3.43 *
Time × Conc	F_9,__48_ = 1.75	F_9,__48_ = 10.39 **	F_9,__48_ = 1.83	F_9,__48_ = 2.57 *	F_9,__48_ = 1.69

Significance levels are indicated by * and ** repsenting *p* < 0.05 and *p* < 0.01, respectively.

#### 2.1.3. Effects of Cd on MDA Content

**Figure 2:**
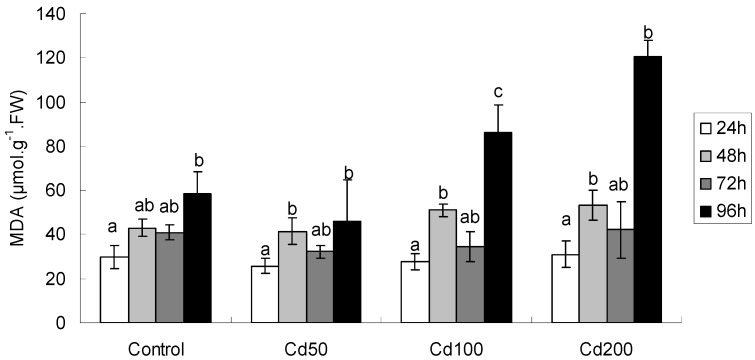
MDA concentration in germinating soybean seeds after different periods of exposure. All values are mean ± SD (n = 4). Values followed by lower case letters are significant at *p* < 0.05.

Oxidative stress was observed in germinating soybean seeds due to Cd treatment. MDA, an indicator of lipid peroxidation, showed exposure time-and dosage-dependent fluctuations (Table 2; Figure 2). In general, MDA concentration was lower at Cd50, and after a reduction at Cd100, it increased at Cd200 again as compared to control (Figure 2). An interesting finding was that MDA concentration increased with prolonged exposure time at control treatment although it was only significantly increased after 96 h exposure as compared to control. In addition, MDA concentration at Cd200 was significantly higher than that of control, Cd50 and Cd100 (F = 26.7, *p* < 0.001) after 96 h exposure. MDA concentration at Cd50 and Cd100 also differed significantly from each other (F = 24.8, *p* = 0.003).

ANOVA analysis indicated that at all Cd concentrations examined, MDA concentrations were correlated positively with protracted exposure time. At Cd50, Cd100 and Cd200, the coefficients (r) and *p* values were 0.82, <0.001, 0.76, 0.001 and 0.66, 0.01, respectively.

#### 2.1.4. Effects of Cd on GSH/hGSH Concentration

**Figure 3:**
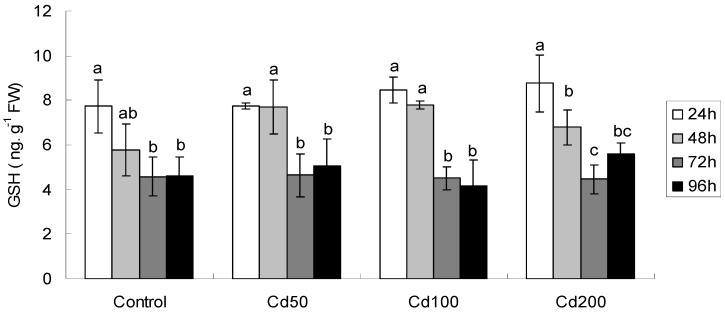
GSH/hGSH concentration in germinating soybean seeds after different periods of exposure. All values are mean ± SD (n = 4). Values followed by lower case letters are significant at *p* < 0.05.

The results suggested a declining tendency of GSH/hGSH concentration with protracted exposure time at any of these treatments (Figure 3, Table 2). In particular, at control, Cd50 and Cd100 treatments, GSH/hGSH concentrations were all significantly lower than those at control after 48 h and 96 h exposure, while the differences were insignificant after 24 h exposure. At Cd200, GSH/hGSH concentrations were all decreased significantly compared to control at any exposure time (F = 18.89, *p* < 0.001). Moreover, ANOVA analysis also suggested a significantly negative correlation between Cd concentration and exposure time. The correlation coefficients (r) and p values at Cd50, Cd100 and Cd200 were −0.74, 0.001, −0.9, <0.001 and −0.75, 0.001, respectively. 

#### 2.1.5. Effects of Cd on Proline Content

**Figure 4:**
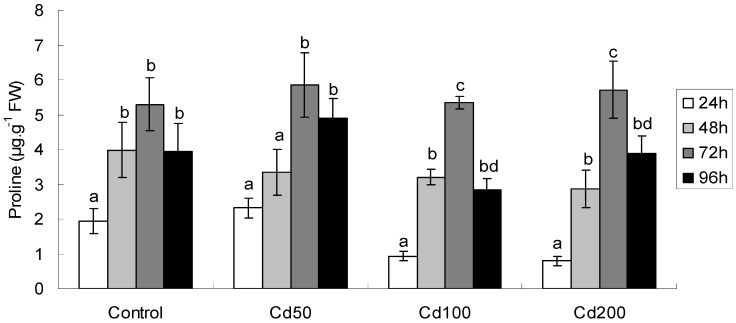
Proline concentration germinating soybean seeds after different periods of exposure. All values are mean ±SD (n = 4). Values followed by lower case letters are significant at *p* < 0.05.

Proline content in soybean seeds increased with exposure time at any of these treatments used in the experiment (Table 2, Figure 4). An obvious tendency was that proline content in seeds increased after 24, 48 and 72 h exposure, but decreased thereafter. Moreover, after 48 h exposure proline contents at any Cd treatment increased significantly as compared to that after 24 h exposure. In addition, a dosage-dependent increase in proline content was observed after 24 h-and 96 h-exposure (F = 22.6, *p* < 0.001 and F = 53.2, *p* < 0.001 for 24 h- and 96 h-exposure, respectively). An exposure time and dosage interaction was also observed for proline content (Table 2). The coefficients (r) and *p* values at Cd50, Cd100 and Cd200 were 0.78, <0.001, 0.68, 0.007, and 0.76, 0.001, respectively.

#### 2.1.6. Effects of Cd on GST Activity

**Figure 5:**
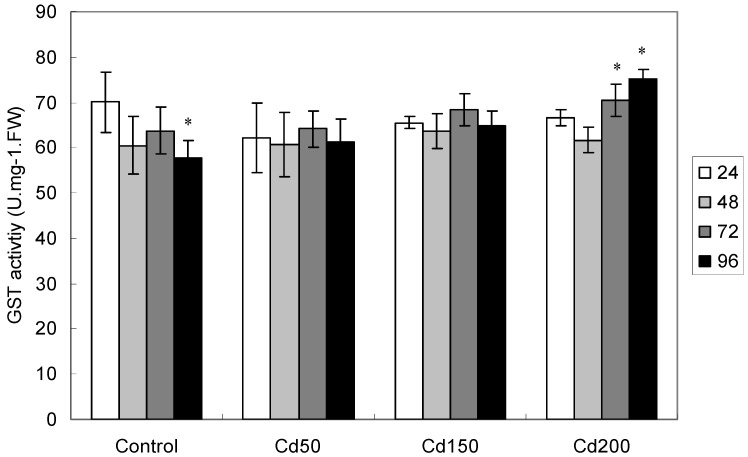
GST concentration germinating soybean seeds after different periods of exposure. All values are mean ± SD (n = 4). * means significance at *p* < 0.05 as compared to control as compared to control. (the same below).

Dosage-and exposure time-dependent changes of GST activity were found in the present study (Table 2). In control group, GST activity decreased significantly with increasing exposure time, and it was reduced by 17.5% after 96 h exposure as compared to that at 24 h (Figure 5). GST activity however increased with increasing exposure time at Cd200, and it was increased by 5.9% and 7.7% after 72 and 96 h exposure as compared to that at 24 h exposure (Figure 5). No significant difference was found at treatment Cd50 and Cd100 with increasing exposure time (Figure 5).

#### 2.1.7. Effects of Dosage, Exposure Time and Dosage × Exposure Interaction

Two-way ANOVA indicated that exposure time affected significantly all these parameters measured in the experiment (Table 2). Cd concentration impacted markedly MDA content and proline content as well as GST activity, while the interaction of exposure time and cadmium concentration has a profound effect on MDA and proline contents (Table 2). Significant difference was also observed between treatments (different cadmium concentration) for MDA content and cadmium concentration in germinating seeds. Similarly, significant difference was also found between exposure time in all these parameters measured except for GST activity.

### 2.2. Discussion

Cadmium is a well known non-essential element that causes oxidative stress in plants. Most attention so far has been paid to the effects of Cd exposure on seedlings or adult plants, and we know little about the oxidative stress and antioxidant mechanisms in germinating seeds. The present study indicated that, challenged by Cd, MDA content and proline level of germinating soybean seeds responded in a exposure time, treatment concentration and exposure time × treatment concentration interacting manner; at Cd200 GST responded to both the exposure time and treatment concentration, while FRAP and GSH responded to exposure time (Table 2). Although soybean seeds germinated under all treatment concentrations, seed germination at higher concentration, *i*.*e*., Cd100 and Cd200, was delayed (Yang *et* *al*., personal observation). In addition, Cd concentration in germinating seeds increased with increasing exposure time and treatment concentration (Table 1).

FRAP is a semi-quantitative method developed to quantify the enzymatic and non-enzymatic antioxidants present in plants and animals [12,13,14]. An exposure time-dependent increasing in FRAP concentration was observed in our experiment among all treatments, but the increasing tendency was depressed after 48 h of exposure though insignificant; the interaction between exposure time and treatment concentration was not found (Table 2; Figure 1). In germinating mustard seeds exposed to the same Cd concentration, Szőllősi *et* *al*., [15] observed that FRAP responded markedly to exposure time, treatment concentration and their interactions. Szőllősi *et* *al*. also found that at Cd50, FRAP activities tended to decrease during 12–24 h exposure, and increase later on, though this effect was insignificant [15], but at Cd100 and Cd200, FRAP activities decreased significantly during 12–96 h exposure. The general declining tendency of FRAP’s response to Cd exposure is similar to the results of present study. The difference of sensitivity of FRAP toward Cd exposure may be species-specific.

Malondialdehyde (MDA) is a product of lipid peroxidation and the most honest marker of oxidative stress in plants in response to stress conditions. Profound decrease in lipid peroxidation has been observed in cadmium-treated germinating seeds of *Brassica* *juncea* L. [15]. As expected, MDA content increased significantly with increasing Cd concentration and prolonged exposure time (Figure 2; Table 2). The highest MDA content was observed at Cd200 and 96-h exposure (Figure 2), suggesting that higher Cd levels induced lipid peroxidation, resulting in irreversible damage to membrane. A possible reason may be that in the context of higher Cd concentration and longer exposure time, the antioxidant load e.g., proline and GSH/hGSH content, is being depleted (Figure 3 and Figure 4), rendering less antioxidative capacity [16,17,18,19]. Seed coat also functions as a barrier to prevent the entry of Cd into germinating seeds [20]. But with prolonging exposure time and increasing Cd concentration, seeds imbibe Cd-containing solution, expand and break, more Cd enters the seeds, causing cytotoxicity and leading to peroxidation of lipid membrane (Figure 2). Similar findings were also observed in tomato seedlings [21] and a Cd-hyperaccumulator, *Solanum* *nigrum* L. [22] challenged by Cd exposure. However, the general changing tendency of MDA in our experiment was increasing with increasing Cd concentration and exposure time, which was opposite to that reported by Szőllősi *et* *al*. [15] in germinating Indian mustard seeds. The reasons behind the differences between both research remains to be elucidated.

GSH plays an important role in regulating the antioxidative mechanism and redox homeostasis of plant cells [16,17,20,21]. Homologous GSHs (hGSHs) are usually found alongside GSH in many legumes, which indicate the signaling in optimal and stress conditions [23]. GSH/hGSH content decreased slightly after 48 h exposure, but it declined significantly after 72 h and 96 h exposure (Figure 3). The treatment concentration of Cd affected insignificantly GSH content (Table 2). Similarly, by using germinating pea seeds as study model, Smiri *et* *al*. [6] also observed a two-fold lower GSH/hGSH levels in Cd-treated germinating pea seeds. The subsequent depletion of GSH (Figure 3) may be attributed to the involvement of GSH in the synthesis of phytochelatins induced by Cd [16,17] and the formation of Cd-GSH complexes [16]. Challenged by 100 mg/L Cd, the GSH content in germinating mustard seeds increased markedly after 24 h exposure, but decreased afterwards [15]. By using the same Cd concentration, depressed GSH content was observed after 48 h exposure in the present study (Figure 3), and Gallego *et* *al*. [24] observed a depletion of GSH pool after 96 h exposure in sunflower seeds treated by 100 and 200 μM Cd as compared to the control seeds.

It has been demonstrated that plants accumulate proline to counteract osmotic stress. Proline accumulation is not only an indicator of abiotic stress, but also an important protecting agent against heavy metal stress [23,24] since exogenously proline can improve the antioxidative capacity and confer cultured tobacco cell’s tolerance to Cd exposure [25]. Free proline has been found to chelate Cd ion in plants by forming non-toxic Cd-proline complex [26]. In addition, accumulating results also suggested that proline accumulation in response to heavy metal may be related to its antioxidative nature [27,28]. An exposure time- and treatment concentration-dependent production of proline was observed in our experiment (Table 2; Figure 4), and there was also exposure time × dosage interaction for proline content (Table 2), suggesting that with increasing Cd concentration and protracted exposure time, the antioxidant mechanisms in germinating seeds were potentiated.

Glutathione-S-transferases can catalyze glutathione-dependent isomerization and the reduction of toxic reactive oxygen species [29] by acting as glutathione peroxidase [30]. Dixit *et* *al*. also observed a significant increase of GST activity in pea plants subjected to Cd treatment [31]. The highest increase in GST activity was observed in pea plants after three-day exposure to 40μM Cd, suggesting that GST might have played some role in the metal detoxification process [31,32]. A similar change was also observed in our experiments. After Cd200 treatment, the highest GST activity was observed after 72 h and 96 h exposure (Figure 5). In germinating Indian mustard seeds, however, Szőllősi *et* *al*. found an initial increase, but a later on declining trend of GST activity with the same treatment [15]. In *Phragmites* *australis* plants treated chronically with 50 μM CdSO_4_, enhanced GST activities were found in leaves, roots and stolons as compared to control [33]. Whether Cd-induced increase in GST activity is a detoxification response is the subject of future study.

## 3. Experimental Section

### 3.1. Seed Germination and Cadmium Exposure

Soybean (*Glycine* *max*. L.) seeds with uniform size were sterilized in 10% NaClO for 10 min and then were germinated in Petri dishes containing vermiculite. The vermiculite was saturated with different concentrations of CdCl_2_·2.5H_2_O solution prepared with distilled water. Cd concentrations used in this experiment were 0 mg/L, 50 mg/L, 100 mg/L and 200 mg/L, hereafter indicated as Cd0, Cd50, Cd100 and Cd200, respectively. Twenty soybean seeds were germinated for each treatment. The Petri dishes were then kept in dark for 24, 48, 72 and 96 h according to natural temperature rhythm in July in Wuhu, China. There were four replicates for each treatment.

### 3.2. Cd Content in Seeds

Cadmium content was determined by atomic absorption spectrophotometer (AAS, Shimazadu, AA-6300C, Kyoto, Japan). Dried seeds were homogenized and 0.20 g seed powder was digested with mixture of HClO_4_ and HNO_3_ (v/v = 1:4) at 200 °C until white smoke disappeared. The digested colorless liquid was washed by 2 mL diluted HNO_3_ (distilled water/concentrated HNO_3_ = 1:1) and then by distilled water for three times. The liquid collected was then transferred to 25 mL volumetric flasks. Four replicates were taken for each concentration and exposure time. Values of Cd content were expressed as μg/g dry weight.

### 3.3. Samples Preparation

Germinating seeds (1.0 g) were homogenized with 4 mL cold sodium phosphate butter solution (0.1 M, pH 7.6) containing 0.1 mM EDTA, 0.5% (v/v) Triton X-100 and 0.5% (w/v) PVPP and centrifuged at 10,000 g for 15 min at 4 °C. The supernatant was used for the determination of oxidants and antioxidants as well as proline content. All samples are four replicates.

### 3.4. Determination of Total Antioxidant Capacity

A simple and quick method called ferric reducing ability of plasma (FRAP) [34] was used for the determination of the antioxidant power of the extracts. FRAP reagent was prepared by mixing 10 mM 2,4,6-tripyridyl triazine (TPTZ; Aladdin, Shanghai, China), 20 mM ferric chloride (FeCl_3_·6H_2_O; BBI) and sodium acetate (BBI) buffer (pH 3.6) at a ratio of 1:1:10 (v/v/v). Reaction mixture contains 50 μL seed extract and 2.95 mL FRAP reagent and was incubated at 37 °C for five minutes. The absorbance was read spectrophotometrically (P General, TU-180AP, Beijing, China) at 593 nm. The reaction mixture containing 3 mL FRAP serves as control. The total antioxidant capacity was expressed as units of μmol/mL seed extract.

### 3.5. Determination of Lipid Peroxidation

Malonyldialdehyde (MDA) is one of the end products of lip peroxidation and it was determined according to Szőllősi *et* *al*. [15] with some modification. Seed extract (150 μL) was mixed with a mixture of 10% (w/v) trichloroacetic acid (Aladdin, AR) and 0.5% (w/v) thiobarbituric acid (BBI, AR). The mixture was heated for 15 min at 96 °C in water bath and then cooled down in cold water to room temperature. The resulting solution was then centrifuged for 5 min at 4,000 g. Absorbance of the supernatant was read at 532 nm and 600 nm, respectively and MDA content was calculated using an extinction coefficient of 155 mM^−1^ cm^−1^. The value of MDA was expressed as μmol.g^−1^ FW. 

### 3.6. Glutathione/Homoglutathione (GSH/hGSH)

The non-proteinic thiols (glutathione and functionally homologous glutathione, GSH/hGSH hereafter) was quantified spectrophotometrically by utilizing the Ellman’s regent [35]. Briefly, 250 μL seed extract was added to 3.0 mL reaction mixture containing 2.6 mL sodium phosphate buffer solution (0.1 M, pH 7.6) and 150 μL DTNB (10 mM). The absorbance at 340 nm was read after five-minute incubation at 30 °C. GSH content was expressed as ng·g^−1^ FW.

### 3.7. Proline Content

Proline content was estimated according to Ringel *et* *al*. [36] with some modification. Seed extract (0.5 mL) was mixed with 3% (w/v) aqueous sulfosalicylic acid (BBI, AR), 1 mL acetic acid (BBI, AR) and 2.5% acidic ninhydrin reagent. The reaction mixture was heated in boiling water for 30 min and proline was extracted with toluene. Absorbance of the supernatant was read at 520 nm, and proline content was calibrated by a standard curve. Unit of proline content is μg·g^−1^ FW.

### 3.8. Determination of GST Activity

GST activity was measured following the method of Cui *et* *al*. [37] with small modification. Briefly, 10 μL seed extract was mixed with 2.95 mL sodium phosphate buffer (0.1 M, pH 7.4) containing 10 μM reduced glutathione (Sigma-Aldrich, AR). The reaction was started by adding 40 μL 1-chloro-2,4-dinithrobenzene (BBI, AR). The change of absorbance was followed spectrophotometrically at 340 nm for 2.5 min (P-general, TU1810AP, Beijing, China). One unit (U) is the amount of enzyme producing 1 μmol conjugate produced in one minute. The activity was calculated from the extinction coefficient of 9.6 mM^−1^ cm^−1^ and expressed as U mg^−1^ FW.

### 3.9. Statistic Analysis

All data were analyzed using SPSS 15.0 software package. Two-way analysis of variance (ANOVA) was applied to investigate the effects of Cd treatment and exposure time on the parameters examined. Data are expressed as mean values ± standard deviation (SD) and calculated by fresh weight. Cd content in germinating soybean seeds was calculated by dry weight.

## 4. Conclusions

Based on the present work, high concentrations of Cd caused oxidative stress in the early stage of plant development, seed germination. In response, the content of proline, a non-enzymatic antioxidant, increased initially then attenuated later on, while that of other non-enzymatic antioxidants, GSH and FARP, kept declining. The activity of the enzymatic antioxidant, GST, kept increasing, which was contrary to one of its substrates, GSH. Taken together, MDA is good bio-indicator for monitoring the phytotoxicity of Cd in germinating seeds, at least for germinating soybean seeds, but care should be taken when using proline content as a biomarker because it increases initially but attenuates when the exposure time is rather long, an adaptive-like phenomenon. Further work is required to gain insight into the antioxidant mechanisms of adult soybean plants in response to abiotic stress factors like heavy metals.
